# LncRNA TUG1 regulates ApoM to promote atherosclerosis progression through miR‐92a/FXR1 axis

**DOI:** 10.1111/jcmm.15521

**Published:** 2020-06-28

**Authors:** Liu Yang, Tie Li

**Affiliations:** ^1^ International Medical Center Geriatric Department National Clinical Research Center for Geriatric Diseases Xiangya Hospital of Central South University Changsha China; ^2^ Department of Cardiology Changsha Central Hospital Changsha China

**Keywords:** ApoM, atherosclerosis, cholesterol efflux, FXR1, LncRNA, miR‐92a, TUG1

## Abstract

This study aims to explore the possible mechanism of TUG1 regulating ApoM in AS. To this end, expression levels of TUG1 and ApoM were measured in high fat dieted C57BL/6J mice, normal dieted C57BL/6J mice, ob/ob mice and db/db mice. LV‐TUG1 or sh‐TUG1 was injected into C57BL/6J mice before isolating peritoneal macrophages to measure cholesterol efflux (CE) and expression levels of ABCA1, ABCG1 and SR‐BI. Meanwhile, CE in RAW264.7 cells was also measured after cell transfection. Dual luciferase reporter assay and anti‐AGO2 RIP were applied to verify the relationship among TUG1, FXR1 and miR‐92a. Total cholesterol (TC), triglyceride (TG), low‐density lipoprotein cholesterin (LDL‐C), high‐density lipoprotein cholesterol (HDL‐C) as well as expressions of inflammatory cytokines (TNF‐α, IL‐1β and IL‐6) in plasma were measured. Knock‐down or expressed TUG1, FXR1 or miR‐92a in NCTC 1469 cells or in ApoE−/− AS mice to determine the alteration on ApoM and plaque size. TUG1 was highly expressed while ApoM was down‐regulated in high fat dieted C57BL/6J mice, b/ob and db/db mice. Overexpression of TUG1 could reduce the expression of ApoM, ABCA1 and ABCG1 in addition to slowing down CE rate. Reversed expression pattern was found in cells with knock‐down of TUG1. TUG1 can compete with FXR1 to bind miR‐92a. FXR1 negatively target ApoM. Overexpression of TUG1 in ApoE−/− mice can increase plaque size and enhance macrophage contents accordingly. TUG1 can inhibit ApoM in both liver tissues and plasma to inhibit CE through regulating miR‐92a/ FXR1 axis. TUG1 is a promising target for AS treatment.

## 1 INTRODUCTION

Cardiovascular disease (CVD) has gradually surpassed cancer to dominate the highest mortality in Europe, which is responsible for over 3.9 million death per year.^1^ Among all the risk factors of CVD, atherosclerosis (AS) is a paramount leading cause of CVD‐related death among all the population.^2^ AS is characterized by lipid‐laden macrophages within the coronary arteries. Those plaques could accumulate and progress into thrombosis, which consequently block the blood circulation at any moment or lead to fatal consequences such as haemorrhage, rupture and calcification.^3^, ^4^ Nowadays, most applicable therapeutic strategies are preventive and mainly focus on attenuating the formation of thrombus and improving blood lipid profile, yet no treatment can directly target the atherosclerotic lesion.^5^, ^6^ The emergence of next‐generation sequencing facilitates the elucidation of genetic perspective on AS development and progression; therefore, gene target therapy has boosted as a potential field for AS patients.^7^

Long non‐coding RNAs (lncRNAs) have been implied to be involved in many human diseases, including cancers and AS.^8^ Moreover, several lncRNAs are emerged as novel molecular biomarkers for early diagnosis, potential therapeutic targets and prognosis of AS.^9^, ^10^, ^11^ Taurine up‐regulated gene 1 (TUG1) was initially identified as a transcript up‐regulated by taurine, whose aberrant expression has been found in several cancers, including non‐small cell lung cancer, bladder cancer and osteosarcoma.^12^, ^13^, ^14^, ^15^ Additionally, overexpression of TUG1 was reported to hinder the attenuation of tanshinol on oxidized low‐density lipoprotein (ox‐LDL)–induced endothelial cell apoptosis.^16^

Apolipoprotein (apo) M is a typical lipocalin for the lipid sphingosine‐1‐phosphate (S1P). ApoM, by delivering S1P to the S1P (1) receptor on endothelial cells, affects high‐density lipoprotein (HDL) metabolism to exhibit anti‐atherosclerotic functions, such as protection against oxidation and regulation of cholesterol efflux (CE).^17^, ^18^ Furthermore, ApoM is an indispensable constitute for HDL and preβ‐HDL formation to enhance CE.^19^ Macrophage reverse cholesterol transport (RCT) is a protective mechanism of AS which can inhibit accumulation of excessive cholesterol in macrophages, while CE, mediated by three membrane proteins, including ABCA1, ABCG1 and SR‐BI, is the key point for RCT.^20^, ^21^ As far as we known, TUG1 is associated with AS, but the mechanism of TUG1 on AS progression are not fully addressed. Considering the protective role of ApoM in AS, the possibility of TUG1 regulating ApoM in AS also needs to be identified.

MicroRNAs (miRs) have vital roles to play in CVDs, including hypertension and cardiovascular remodelling.^22^ miR‐92a has been emerged as a potential biomarker in AS as miR‐92a silence could promote the expressions of angiogenesis factors.^23^ In current study, we found TUG1 can bind miR‐92a to regulate FXR1. Previous data proved FXR1 can negatively regulate ApoM.^24^ Therefore, we hypothesize that TUG1 regulates ApoM expression mainly through miR‐92a/FXR1 axis to promote AS progression. Collected evidence in this study supported that TUG1 in liver tissues could compete with FXR1 to regulate miR‐92a expression, which results in the down‐regulation of ApoM in both liver tissues and in plasma, consequently leading to inhibition of RCT and deterioration of AS.

## 2 MATERIALS AND METHODS

### 2.1 Subjects

C57BL/6J mice (n = 6, 7 groups), ob/ob mice (leptin receptor–deficient mice, n = 6), db/db mice (diabetic mice, n = 6) and ApoE−/− male mice (n = 6, 4 groups) all purchased from Nanjing Pengsheng Biological Technology Co., Ltd. All the 8‐week aged mice were raised in SPF laboratory at the temperature of 20–25℃ and the humidity of 65%–75% with good ventilation condition. Mice in high fat diet group (HFD group, n = 6) and normal diet group (ND group, n = 6) were fed for 8 weeks. The diets for HFD mice were fat content diet (protein: 20% kcal, fat: 45% kcal, carbohydrate: 35% kcal, energy density: 4.7 kcal/g, cholesterol: 196.5 mg/kg, D12451; Research Diets) while that of ND mice were fat content diet (protein: 20% kcal, fat: 10% kcal, carbohydrate: 70% kcal, energy density: 3.82 kcal/g, cholesterol: 51.6 mg/kg, D12450H; Research Diets). This design of experiments was approved by local commitment of Xiangya Hospital of Central South University and was strictly implemented according to institutional guidelines. Experiments were performed in a humanistic way.

### 2.2 Cell culture

Mouse liver cells (NCTC 1469), human embryonic kidney cells (HEK 293T) and RAW264.7 cells were purchased from American Type Culture Collection (ATCC). Cells were cultured with DMEM culture medium (Gibco) which contains 10% FBS and 1% penicillin/streptomycin in 5% CO_2_ and 37°C incubator.

### 2.3 Quantitative reverse transcription polymerase chain reaction (qRT‐PCR)

Total RNAs from mouse liver tissues or macrophages were isolated using TRIzol (Invitrogen) and subjected to reverse transcript using a reverse transcript kit (TaKaRa) according to the instructions indicated in the kit. LightCycler 480 (Roche) was used for mRNA detection. The conditions were set based on the instructions on the PCR kit (SYBR Green Mix; Roche Diagnostics). The PCR amplification was conducted based on following conditions: 95℃ for 10 seconds, 45 cycles of 95℃ for 5 seconds, 60℃ for 10 seconds and 72℃ for 10 seconds, and extension at 72℃ for 5 minutes. Each reaction for PCR was performed in triplicate. U6 or GAPDH was used as internal control. The mRNA expressions were calculated based on 2‐ΔΔCt method.^25^ ΔΔCt = experimental group (Ct_target gene_ − Ct _internal control_) − Control group (Ct_target gene_ − Ct_internal control_). The primer sequences for genes are listed in Table 1.

**TABLE 1:** Primer sequences for genes in quantitative reverse transcription polymerase chain reaction

Name of primer	Sequences
miR‐92a‐F	GACGUCCGGCCCUGUUCA
miR‐92a‐R	GCAGGGTCCGAGGTATTC
U6‐F	CTCTCGCTTCGGCAGCACA
U6‐R	ACGCTTCACGAATTTGCGT
TUG1‐F	GGGGACCAACCAAGGCAATA
TUG1‐R	ACACCGGGGCATTAATGTGT
ApoM‐F	GGCCAAAAAGGCTCCCTAGT
ApoM‐R	ACTTCTCTGGAGGGTGTGGT
FXR1‐F	GAGTGTGTGTGGTTGCATTGT
FXR1‐R	GAGTGCCCAAGATAGCAGCC
ABCA1‐F	GTGGCTTCGGAGTGTCAAGA
ABCA1‐R	AAACCACTCGCACACATTGC
ABCG1‐F	GCCCGCCGACTCATTATGTA
ABCG1‐R	AGGATGGGGAGCAGCTTAGA
SR‐BI‐F	TCGCATTCACCATCTGGCTT
SR‐BI‐R	TGTGTGACTTCCACGAGAGC
GAPDH‐F	GCAAGGATGCTGGCGTAATG
GAPDH‐R	TACGCGTAGGGGTTTGACAC

Abbreviations: F, forward; R, reverse.

### 2.4 Western blot

Mouse liver tissues or macrophages were lysed using RIPA lysis buffer (beyotime) to obtain the protein sample. After the protein concentration was measured using a BCA kit (beyotime), certain volume of protein was mixed with loading buffer (beyotime) for boiling water bath for 3 minutes for degeneration. The proteins were subjected to electrophoresis at 80 V for 30 minutes and then at 120 V for 1–2 hours. Membrane was transferred at a current of 300 mA for 60 minutes and then washed in washing buffer for 1–2 minutes before blocking at room temperature for 1 hou or at 4℃ for overnight. The membranes were incubated with one of the primary antibodies against GAPDH (ab181602, 1:10 000), ApoM (ab85695, 1:1000), FXR1 (ab129089, 1:1000), ABCA1 (ab18180, 1:200), ABCG1 (ab52617, 1:1000) and SR‐BI (ab217318, 1:2000) (Abcam) at shaking bed at room temperature for 1 hour before washing for 3 × 10 minutes. After that, the membranes were incubated with horseradish peroxidase–labelled goat anti‐rabbit IgG (1:5000; Beijing ComWin Biotech Co., Ltd) for 1 hour and washed for 3 × 10 minutes. The membranes were observed under a chemiluminescence imaging system (Bio‐rad) after development solution was added.

### 2.5 Knock‐down or overexpression of TUG1

TUG1 lentivirus overexpression vector (LV‐TUG1), TUG1 knock‐down vector (sh‐TUG1), miR‐92a mimic (50 nM), miR‐92a inhibitor (50 nM), FXR1 lentivirus overexpression vector (LV‐FXR1) and their negative controls were obtained from Shanghai GenePharma Co., Ltd. Cell transfection for NCTC 1469 cells and RAW264.7 cells were performed using Lipofectamine 2000 regent (Invitrogen) based on the manual. Knock‐down or overexpression of TUG1 in mice was achieved through tail intravenous injection of LV vector (50 µL) for a week.

### 2.6 CE rate

Peritoneal macrophages were isolated from mice in each group. RAW264.7 cells were treated with 50 mg/L ox‐LDL. After cell transfection of LV‐TUG1 or sh‐TUG1 for 12 hours, the culture medium for cell culture was removed and replaced with DMEM culture medium containing 0.2% BSA. Cells were then incubated with 1 mCi/L [^3^H] cholesterol at 37℃ for 24 hours. After that, cells were washed in PBS for twice to remove the unabsorbed [^3^H] cholesterol and cultured with DMEM medium containing 0.2% BSA at 37℃. About 4 hours later, cells were washed in PBS for twice and re‐suspended in DMEM medium containing 10 mg/L ApoA‐I or 50 mg/L HDL at 37℃ for 4 hours. Culture medium and cells were collected to measure the radioactivity of [^3^H] cholesterol using Liquid scintillation counting method. The CE rate = [^3^H] in culture medium/ [^3^H] in cells and in culture medium.

### 2.7 Dual luciferase reporter assay

The binding sites of TUG1 and miR‐92a, and that of miR‐92a and FXR1 were predicted by starBase (http://starbase.sysu.edu.cn/). The wide and mutant sequences were designed accordingly and named as wt‐TUG1, mut‐TUG1, wt‐FXR1 or mut‐FXR1. The sequences inserted into pGL3‐Basic and co‐transfected with miR‐92a mimic (30 nM) or its negative control into HEK293T cells. The firefly luciferase activity and Renilla luciferase activity in each group were measured. The Renilla luciferase activity was used as internal control. Luciferase activity = Firefly luciferase activity/Renilla luciferase activity.

### 2.8 RIP

Cells in each group was collected and washed in PBS for twice before centrifuged at 1500 rpm for 5 minutes. Then, certain volume of RIP lysate was added to fully mix with cells. Beads were re‐suspended. Then, 5 µg of Ago2 antibody (ab32381, 1:50; Abcam) was added while cells in negative control group were incubated with IgG antibody at room temperature for 30 minutes. After that, supernatant was abandoned. Cells were vibrated with 500 µL of RIP Wash Buffer to remove the supernatant for twice. Cells were then added with 500 µL of RIP Wash Buffer for vibration and maintained on ice for further use. The supernatant in magnetic bead tube was removed and 900 µL of RIP Immunoprecipitation Buffer was added in each tube. Cell lysis was centrifuged at 14,000 rpm, 4℃ for 10 minutes. Then, 100 µL of supernatant was added into the bead‐antibody complex to make the final volume of 1 mL for incubation at 4℃ overnight. After transient centrifugation, the supernatant was removed and then 500 µL of RIP Wash Buffer was added for vibration with the supernatant removed. The complex in centrifuge tube was washed for six times. RNA purification: 150 µL of Proteinase K Buffer was added to re‐suspend the bead‐antibody complex at 55℃ for 30 minutes with the supernatant removed. The RNA was extracted for qRT‐PCR.

### 2.9 Dissection of aorta for oil red O staining

Mice were fasted overnight before being sacrificed. Mice were anesthetized by injection of pentobarbital sodium (50 mg/kg). The chest of mouse was opened and the aorta was isolated. The aorta was washed before fixation using 4% paraformaldehyde for 24 hours. The aorta was dissected and fixed using needles with adventitia removed. The aorta was stained in oil red O staining for 5 hours and differentiated under 60% isopropanol. The differentiation liquid shall be replaced for several times till the AS plaques were in red while arterial wall in white. The aortic root was also made into slices for oil red O staining.

### 2.10 Immunohistochemistry

The aortic root slices (4 µm) were baked for 20 minutes before dewax using xylene and washing in distilled water. Slices were then washed in PBS for three times and added with 3% H_2_O_2_ at room temperature for 10 minutes. PBS washing for three times before antigen repair. After that, slices were washed in PBS for three times and blocked with goat serum for 20 minutes at room temperature. Primary antibody of MOMA‐2 (ab33451, 1:50) was added for incubation at 4℃ overnight. PBS washing for three times before slices were incubated with secondary antibody for 1 hours. PBS washing for three times before colour development with DAB for 1–3 minutes. Then, slices were stained with haematoxylin, dehydrate and transparent and sealed.

### 2.11 Measurement on plasma

Total cholesterol (TC), triglyceride (TG), low‐density lipoprotein cholesterin (LDL‐C), high‐density lipoprotein cholesterol (HDL‐C) in plasma were correspondingly determined using detection kits (Nanjing Jiancheng Bioengineering Institute) based on instructions.

### 2.12 Measurement on inflammatory cytokines

Inflammatory cytokines including TNF‐α, IL‐1β and IL‐6 were measured using ELISA kit (R&D Systems) according to instructions.

### 2.13 Statistical analysis

Data were analysed using GraphPad prism7 software. All data were expressed using mean ± standard deviation ( $\bar{x}$ ± SD). Comparison between two groups was achieved through *t* test and data among groups was analysed using one‐way analysis of variance with Dunnett's multiple comparisons test as post hoc test. *P* value of <0.05 was considered to have statistical significance.

## 3 RESULTS

### 3.1 Up‐regulation of TUG1 and down‐regulation of ApoM in liver tissues of HFD dieted mice

**FIGURE 1:**
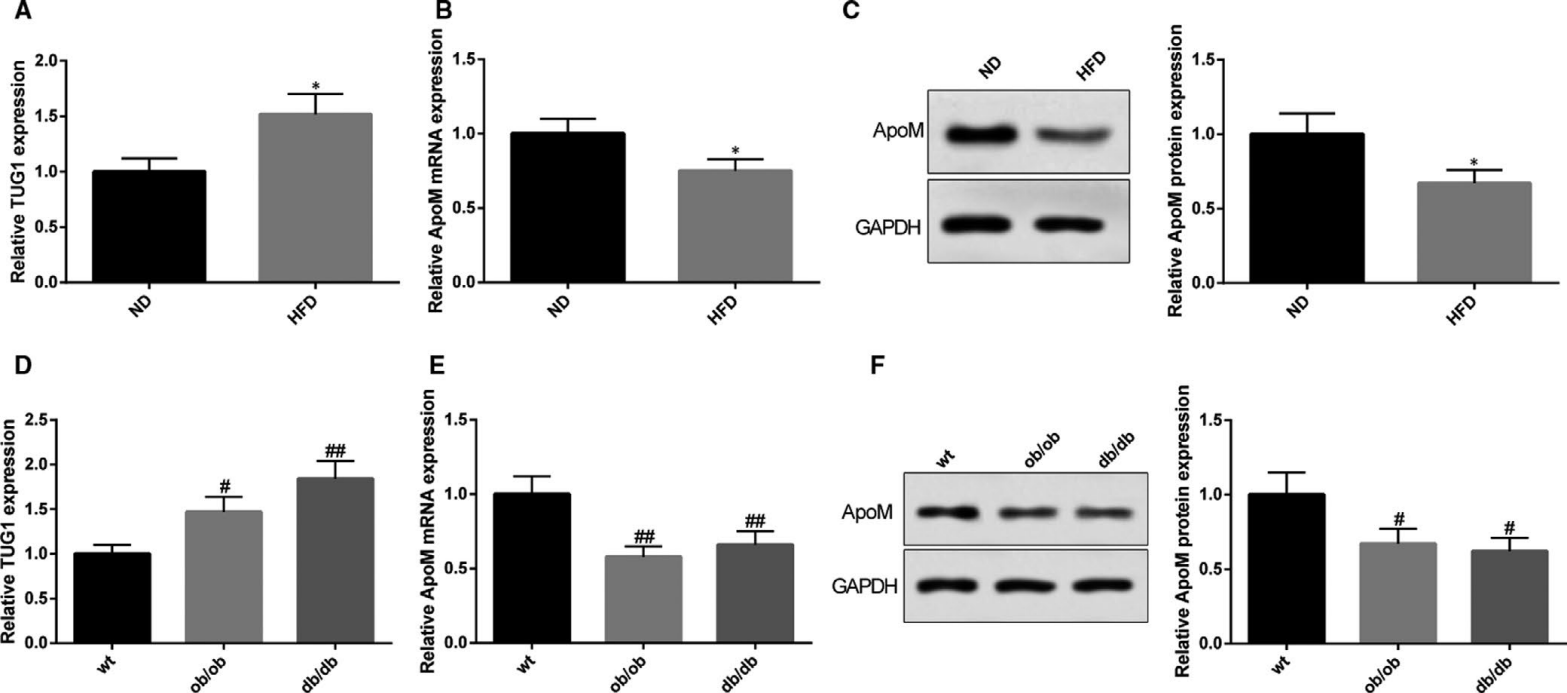
Mice with abnormal lipid metabolism had up‐regulated expression level of TUG1 and down‐regulated expression level of ApoM. TUG1 expressions in liver tissues of mice in both HFD group and ND group were determined using qRT‐PCR (A), n = 6; the mRNA and protein expression levels of ApoM in liver were detected in mice in both HFD group and ND group using qRT‐PCR (B) and Western blot (C), n = 6. Meanwhile, the expression levels of TUG1 (D) and ApoM (E, F) were also measured in liver tissues of wt C57BL/6J mice, ob/ob mice and db/db mice, n = 6. *, compared with ND group, *P* < 0.05; #, compared with wt group, *P* < 0.05; ##, compared with wt group, *P* < 0.01; HFD, high fat diet; ND, normal diet; ob/ob mice, leptin receptor–deficient mice; db/db mice, diabetic mice

The expression levels of TUG1 and ApoM in liver tissues were detected by qRT‐PCR and Western blot. The detection showed that the expression of TUG1 in mice in HFD group is 1.52 times than that of ND group (Figure 1A, *P* < 0.05). The mRNA of ApoM in HFD group is 0.75 times less, while the protein expression of ApoM in HFD group is 0.67 times less than that in ND group (Figure 1B,C, *P* < 0.05). In addition, the expression levels of TUG1 in liver tissues of both ob/ob mice and db/db mice were 1.47 times and 1.84 times than that of wt C57BL/6J mice (Figure 1D, *P* < 0.05). The expression of ApoM was respectively decreased by 0.58 times (mRNA expression) and 0.67 times (protein expression) in ob/ob mice, and 0.66 times (mRNA expression) and 0.62 times (protein expression) in db/db mice, in comparison with that in wt mice (Figure 1E,F, *P* < 0.05). Collectively, TUG1 was highly expressed and ApoM was down‐regulated in liver tissues of mice with abnormal lipid metabolism.

### 3.2 Overexpression of TUG1 inhibits ApoM expression and macrophage CE in C57BL/6J mouse

**FIGURE 2:**
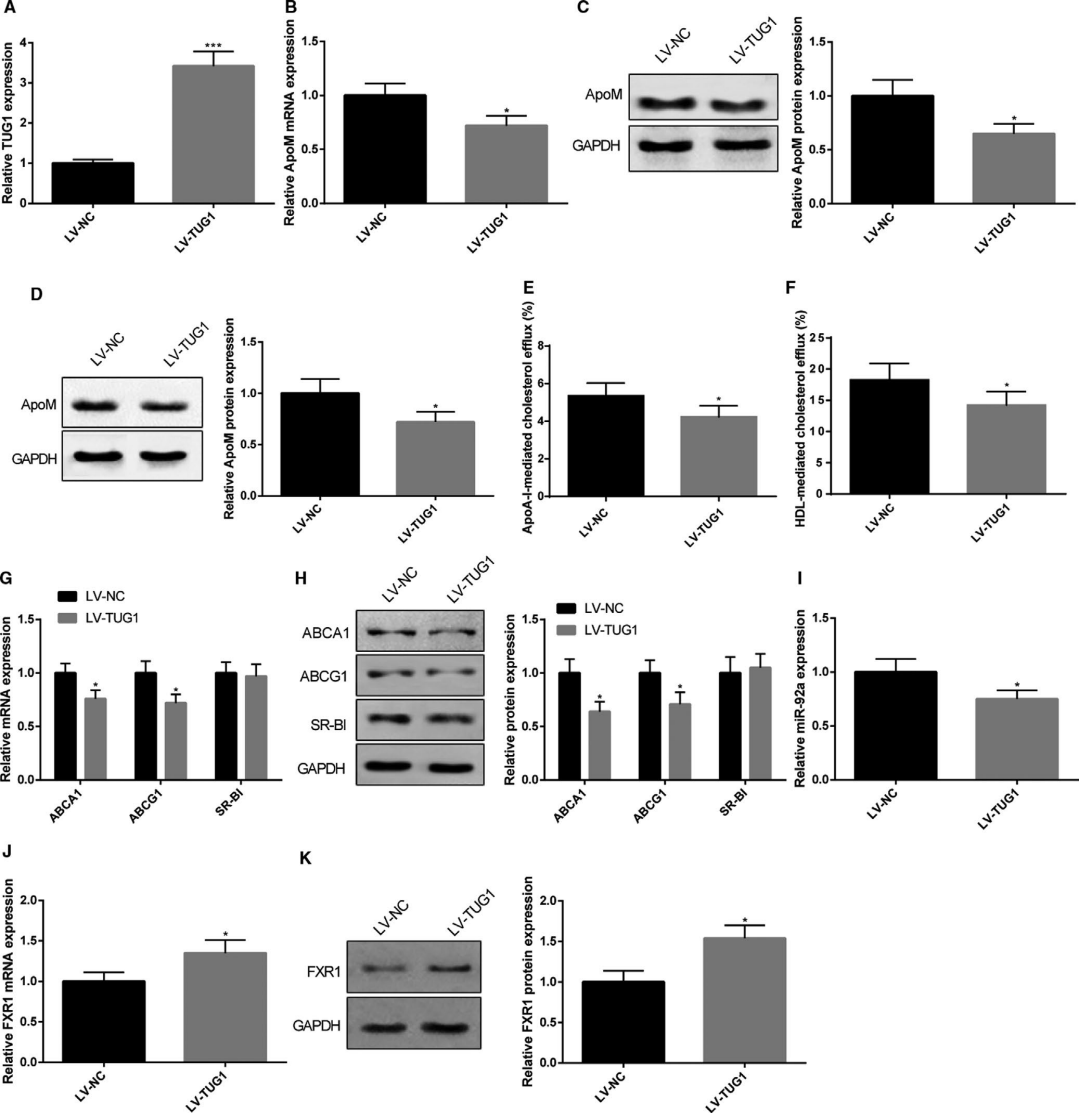
Mice injected with TUG1 had down‐regulated expression levels of ApoM and inhibited macrophage CE rate. C57BL/6J mice were subjected to tail intravenous injection of LV‐TUG1 or LV‐NC before TUG1 and ApoM expression levels in both liver tissues and plasma were determined by qRT‐PCR and Western blot. TUG1 in liver tissues determined by qRT‐PCR (A), n = 6; mRNA and protein expression of ApoM in liver tissues were measured (B, C), n = 6; protein expression of ApoM in plasma was measured (D), n = 6; CE rate was calculated by measuring the radioactivity of [^3^H] cholesterol using liquid scintillation counting method (E, F), n = 6. The alternation of three membrane proteins of RCT, including ABCA1, ABCG1 and SR‐BI in macrophages were determined by qRT‐PCR (G) and Western blot (H), n = 6. qRT‐PCR and Western blot were also applied to measure the expression level of miR‐92a (I) and FXR1 (J, K) in liver tissues, n = 6. *, compared with LV‐NC group, *P* < 0.05; ***, compared with LV‐NC group, *P* < 0.001; CE, cholesterol efflux; RCT, reverse cholesterol transport

After mice were injected with LV‐TUG1, qRT‐PCR and Western blot were applied to detect the expression levels of TUG1 and ApoM in both liver tissues and in plasma. The results showed that TUG1 was up‐regulated 3.42 times in mice in LV‐TUG1 group compared with that in LV‐NC group (Figure 2A, *P* < 0.05). Moreover, LV‐TUG1 could down‐regulate the mRNA expression of ApoM by 0.72 times and the protein expression of ApoM by 0.65 times in liver tissues (Figure 2B,C, *P* < 0.05), while LV‐TUG1 could inhibit the protein expression of ApoM by 0.72 times in plasma (Figure 2D, *P* < 0.05). We then measured the CE rate in mice by comparing the CE rate and expressions of membrane proteins. Mice in LV‐TUG1 group had decreased CE rate (ApoA‐I mediation: 5.35 ± 0.68% vs 4.23 ± 0.59%, HDL mediation: 18.27 ± 2.64% vs 14.27 ± 2.12%, Figure 2E,F, *P* < 0.05), down‐regulated expression levels of ABCA1 and ABCG1 in macrophages than those in LV‐NC group (Figure 2G,H, *P* < 0.05). No significance on expression level of SR‐BI in mice of LV‐TUG1 group and LV‐NC group was noticed (Figure 2G,H). Moreover, mice with injection of LV‐TUG1 had miR‐92a expression decreased by 0.75 times (Figure 2I, *P* < 0.05), FXR1 mRNA increased by 1.35 times and FXR1 protein increased by 1.54 times in liver tissues (Figure 2J,K, *P* < 0.05). Collectively, overexpression of TUG1 could down‐regulate ApoM expression level in both liver and plasma and inhibit macrophage RCT through regulating ABCA1 and ABCG1 pathway.

### 3.3 Knock‐down of TUG1 promotes ApoM expression and macrophage CE in C57BL/6J mouse

**FIGURE 3:**
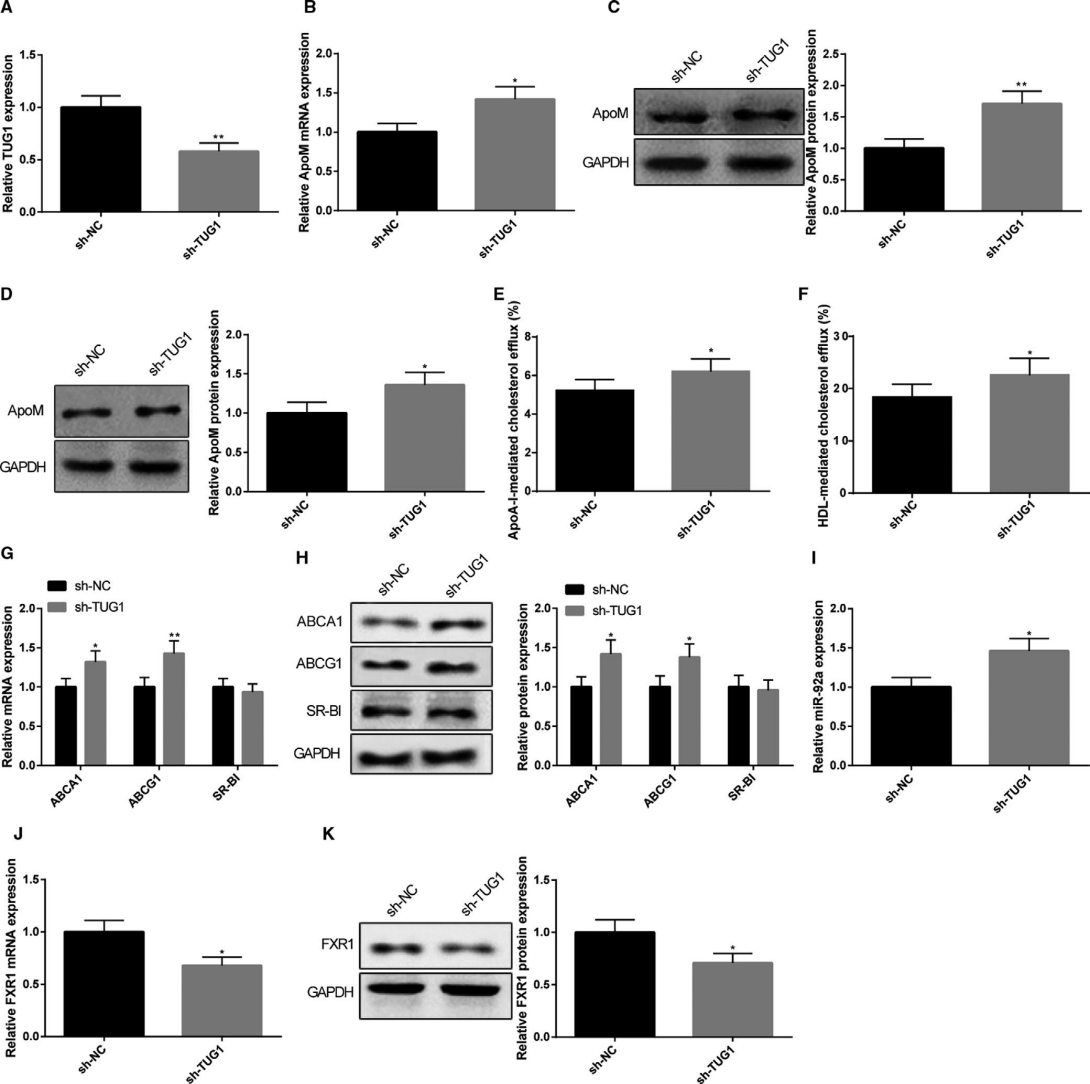
Mice injected with sh‐TUG1 had up‐regulated expression of ApoM and promoted CE rate. C57BL/6J mice were subjected to tail intravenous injection of sh‐TUG1 or L sh‐NC before TUG1 and ApoM expression levels in both liver tissues and plasma were determined by qRT‐PCR and Western blot. TUG1 in liver tissues determined by qRT‐PCR (A), n = 6; mRNA and protein expression of ApoM in liver tissues were measured (B, C), n = 6; protein expression of ApoM in plasma was measured (D), n = 6; CE rate was calculated by measuring the radioactivity of [^3^H] cholesterol using Liquid scintillation counting method (E, F), n = 6. The alternation of three membrane proteins of RCT, including ABCA1, ABCG1 and SR‐BI in macrophages were determined by qRT‐PCR (G) and Western blot (H), n = 6. qRT‐PCR and Western blot were also applied to measure the expression level of miR‐92a (I) and FXR1 (J, K) in liver tissues, n = 6. *, compared with sh‐NC group, *P* < 0.05; **, compared with sh‐NC group, *P* < 0.01; CE, cholesterol efflux; RCT, reverse cholesterol transport

After mice were injected with sh‐TUG1, qRT‐PCR and Western blot were applied to detect the expression levels of TUG1 and ApoM. The results showed that TUG1 was down‐regulated by 0.58 times in mice in sh‐TUG1 group in comparison with that in sh‐NC group (Figure 3A, *P* < 0.05). Moreover, sh‐TUG1 could increase the mRNA expression of ApoM by 1.42 times and the protein expression of ApoM by 1.71 times in the liver tissues (Figure 3B,C, *P* < 0.05), while the protein expression of ApoM was elevated by 1.36 times in plasma (Figure 3D, *P* < 0.05). sh‐TUG1 could also increase CE rate (ApoA‐I mediation: 5.24 ± 0.56% vs 6.23 ± 0.64%, HDL mediation: 18.42 ± 2.45% vs 22.67 ± 3.14%. Figure 3E,F, *P* < 0.05), in addition to elevating the expression levels of ABCA1 and ABCG1 in macrophages of C57BL/6J mice (Figure 3G,H, *P* < 0.05), although no difference was detected regarding the expression level of SR‐BI between the two groups (Figure 3G,H). Meanwhile, we also found miR‐92a expression increased by 1.46 times (Figure 3I, *P* < 0.05), FXR1 mRNA decreased by 0.68 times and FXR1 protein decreased by 0.71 times (Figure 3J,K, *P* < 0.05). Collectively, knock‐down of TUG1 could up‐regulate ApoM expressions in both liver tissues and plasma as well as promoting macrophage CE.

### 3.4 TUG1 suppresses CE in RAW264 cells

**FIGURE 4:**
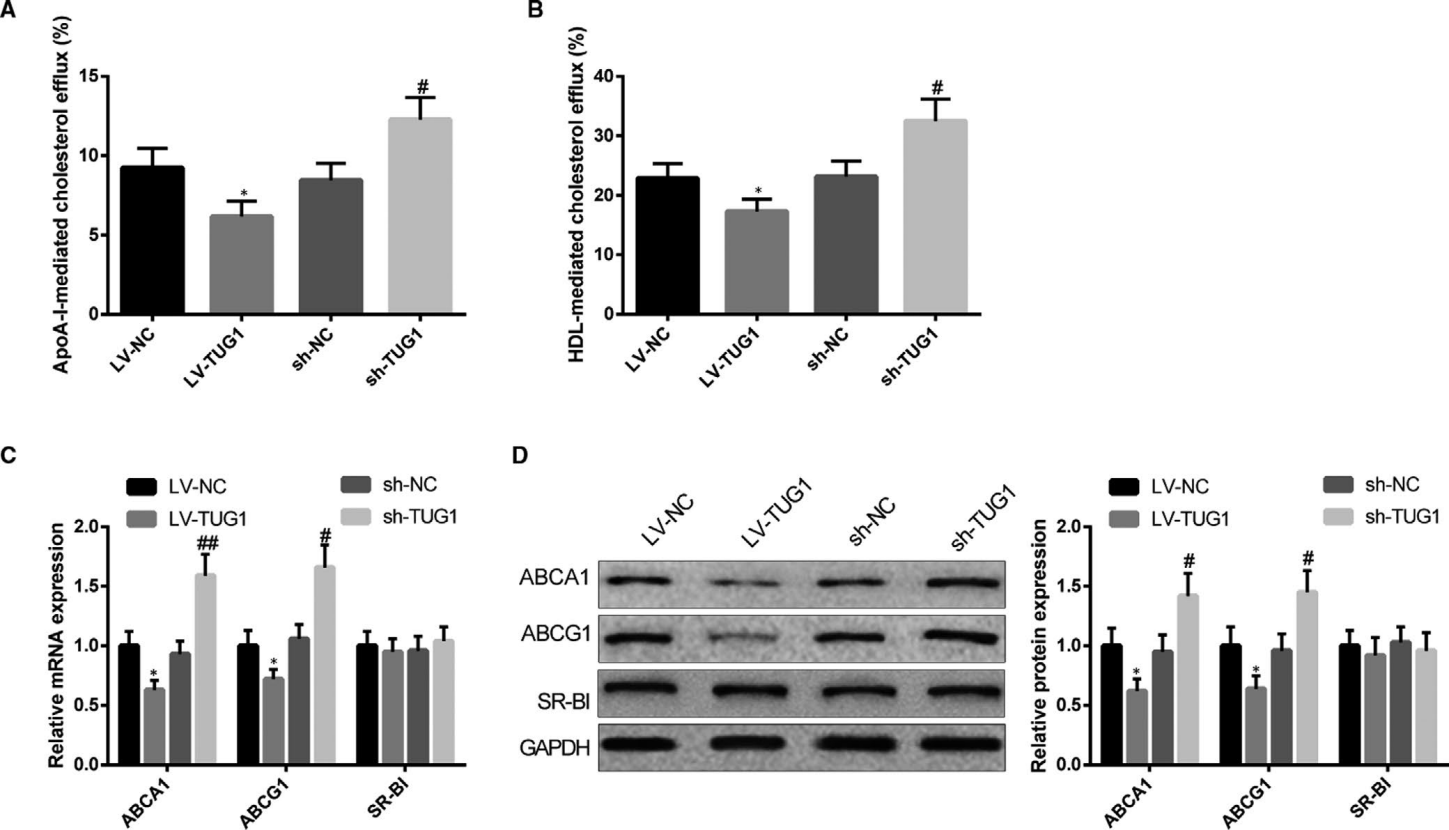
TUG1 suppress CE in RAW264.7 cells. After LV‐TUG1 or sh‐TUG1 was transfected into RAW264.7 cells, radioactivity of [^3^H] cholesterol in RAW264.7 cells and culture medium was measured using liquid scintillation counting method to calculate CE rate (A, B), n = 3; expressions of ABCA1, ABCG1 and SR‐BI in RAW264.7 cells were detected using qRT‐PCR and Western blot (C, D), n = 3. *, compared with LV‐NC group, *P* < 0.05; #, compared with sh‐NC group, *P* < 0.05; ##, compared with sh‐NC group, *P* < 0.01. CE, cholesterol efflux

The effect of TUG1 on CE in RAW264 cells was measured after cells were transfected with TUG1 overexpression or knock‐down plasmid. The detection on CE demonstrated LV‐TUG1 could substantially suppress ApoA‐I and HDL‐mediated CE (ApoA‐I mediation: 9.24 ± 1.22% vs 6.17 ± 0.96%; HDL mediation: 22.84 ± 2.51% vs 17.32 ± 2.02%). On parallel, sh‐TUG1 could increase ApoA‐I and HDL‐mediated CE (ApoA‐I mediation: 8.46 ± 1.06% vs 12.27 ± 1.39%; HDL mediation: 23.15 ± 2.64% vs 32.47 ± 3.67%, Figure 4A,B, *P* < 0.05). Further detection by qRT‐PCR and Western blot showed that LV‐TUG1 could evidently inhibit ABCA1 and ABCG1 expressions in RAW264.7 cells, while sh‐TUG1 could evidently elevate the expressions of ABCA1 and ABCG1 in RAW264.7 cells (Figure 4C,D, *P* < 0.05). Collectively, TUG1 could inhibit CE in RAW264.7 cells, which was in consistent with observations in macrophages.

### 3.5 TUG1 competes with FXR1 to bind miR‐92a

**FIGURE 5:**
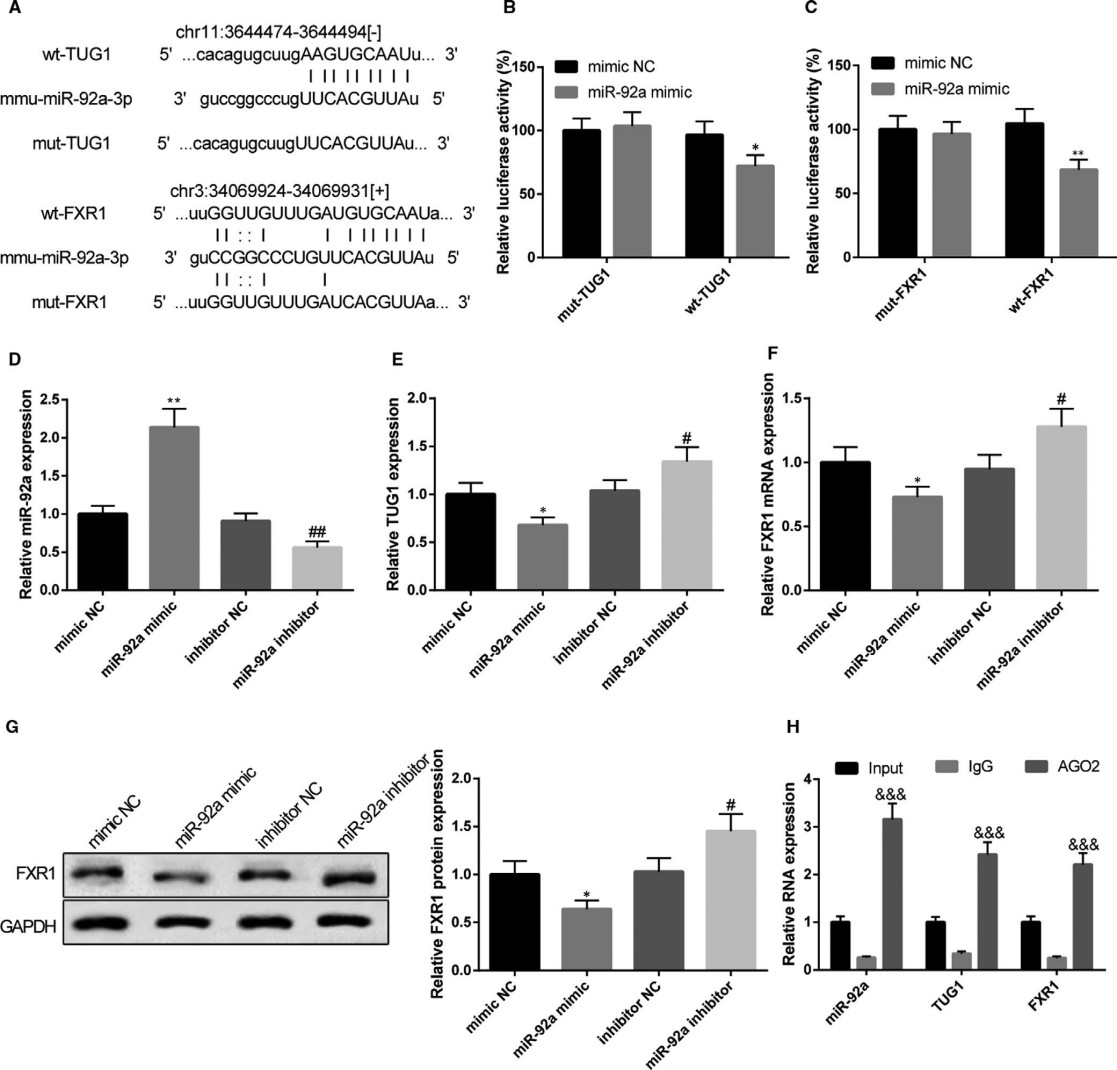
The competitive relationship of TUG1 with FXR1 to bind miR‐92a. Wide type and mutant type of the bind sites predicted by Starbase online software (A). Dual luciferase reporter assay verified the binding of TUG1 with miR‐92a (B), and the target relationship of FXR1 with miR‐92a (C), n = 3. After mouse liver NCTC 1469 cells were transfected with miR‐92a mimic, miR‐92a inhibitor or its negative control, qRT‐PCR and Western blot were applied to measure the expression levels of miR‐92a (D), TUG1 (E) and FXR1 (F, G), n = 3. RIP assay was performed to verify the enrichment of miR‐92a, TUG1 and FXR1 in RNA‐induced silencing complex (RISC) (H), n = 3. *, compared with mimic NC group, *P* < 0.05; **, compared with mimic NC group, *P* < 0.01; #, compared with inhibitor NC group, *P* < 0.05; ##, compared with inhibitor NC group, *P* < 0.01; &&&, compared with IgG group, *P* < 0.001

Starbase (http://starbase.sysu.edu.cn/) software found the binding site of FXR1 with TUG1 and with miR‐92a. Wide type (wt) and mutant type (mut) of binding site were designed for dual luciferase reporter assay (Figure 5A). No difference was revealed in cells inserted with mut‐TUG1 between miR‐92a mimic group and mimic NC group while the luciferase activity in wt‐TUG1 inserted cells in miR‐92a mimic group was much reduced when compared with that in mimic NC group (Figure 5B, *P* < 0.05). As expected, cells inserted with mut‐FXR1 showed no difference in luciferase activity between miR‐92a mimic group and mimic NC group. However, cells inserted with wt‐FXR1 in miR‐92a mimic group reduced luciferase activity in comparison to that in mimic NC group (Figure 5C, *P* < 0.05). Collectively, both TUG1 and FXR1 can directly bind miR‐92a.

Then, we further verified the relationship among the TUG1, FXR1 and miR‐92a in mouse NCTC 1469 cells. RT‐PCR and Western blot after cell transfection found that miR‐92a mimic transfection would evidently up‐regulate miR‐92a expression level (Figure 5D, *P* < 0.05) and down‐regulate both TUG1 (Figure 5E, *P* < 0.05) and FXR1 (Figure 5F,G, *P* < 0.05). Different expression pattern was found in NCTC 1469 cells after transfection of miR‐92a inhibitor. Collectively, it was proved that miR‐92a expression level was negatively associated with those of TUG1 and FXR1. Anti‐AGO2 RIP assay was also performed to verify the enrichment of miR‐92a, TUG1 and FXR1 in RNA‐induced silencing complex (RISC). RIP results demonstrated that AGO2 group found expressions of miR‐92a, TUG1 and FXR1 while IgG group barely found the existence of these three factors (Figure 5H). Taken together, TUG1 competes with FXR1 to bind miR‐92a.

### 3.6 TUG1/miR‐92a/FXR1 axis regulates ApoM in NCTC 1469 cells

**FIGURE 6:**
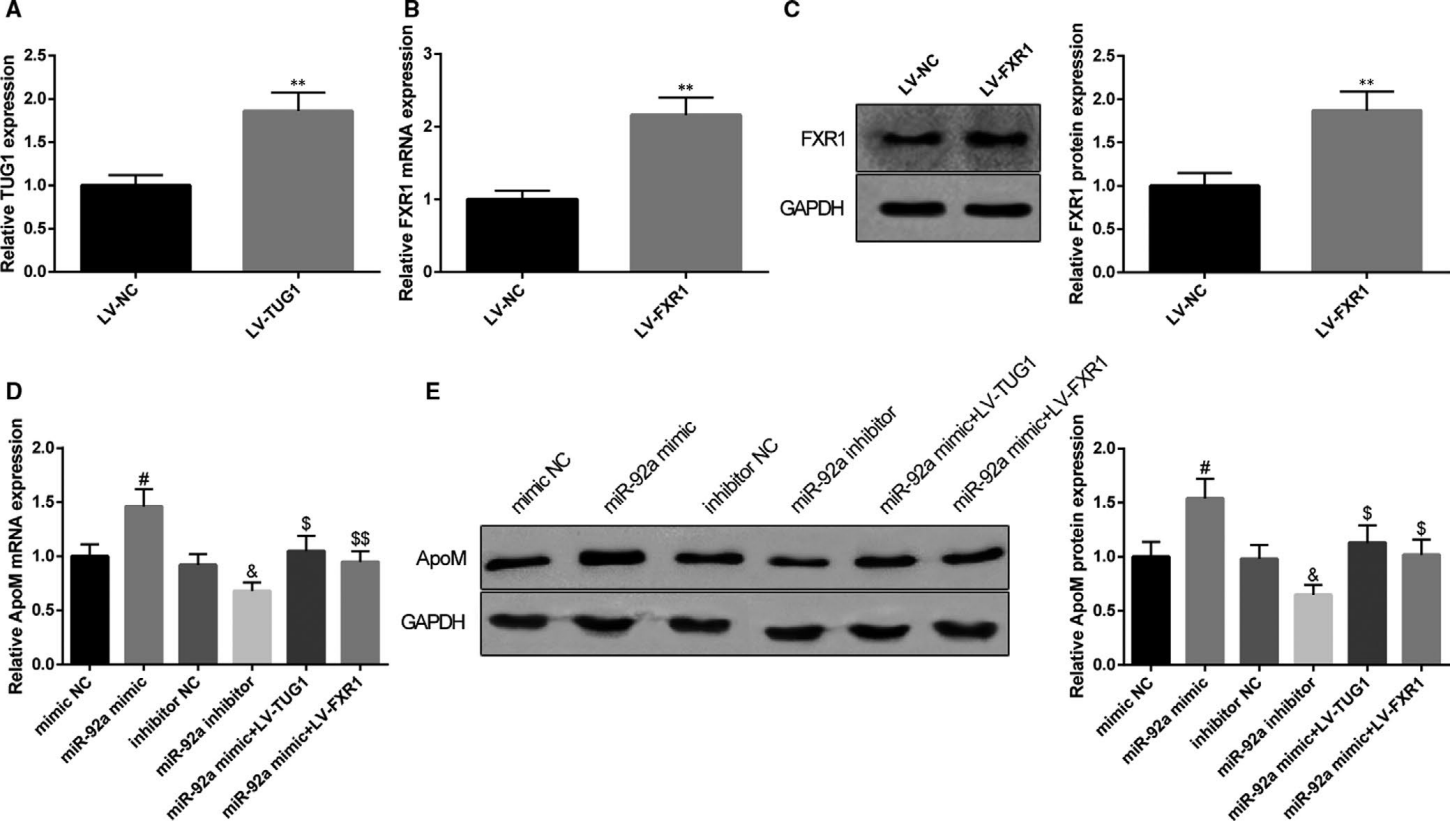
TUG1/miR‐92a/FXR1 axis regulates ApoM expression in NCTC 1469 cells. After NCTC 1469 cells were transfected with LV‐TUG1 or negative control, qRT‐PCR was applied to detect the expression level of TUG1 (A), n = 3. After NCTC 1469 cells were transfected with LV‐FXR1 or negative control, qRT‐PCR and Western blot were applied to detect the expression level of FXR1 (B, C), n = 3. NCTC 1469 cells were subjected to qRT‐PCR and Western blot for measurement of ApoM expressions after being transfected with miR‐92a mimic/miR‐92a inhibitor/negative control or co‐transfected with miR‐92a mimic and LV‐TUG1/LV‐FXR1 (D, E), n = 3. **, compared with LV‐NC group, *P* < 0.01; #, compared with mimic NC group, *P* < 0.05; &, compared with inhibitor NC group, *P* < 0.05; $, compared with miR‐92a mimic group, *P* < 0.05; $$, compared with miR‐92a mimic group, *P* < 0.01

As we proved above that TUG1 in liver tissues of mice negatively regulate ApoM, but the regulatory role of miR‐92a/FXR1 on ApoM remains to be determined. In this regards, we aim to explore the effect of TUG1/miR‐92a/FXR1 on ApoM in mouse liver NCTC 1469 cells. LV‐TUG1 could up‐regulate TUG1 expression in NCTC 1469 cells (Figure 6A, *P* < 0.05) while transfection of LV‐FXR1 could correspondingly up‐regulate FXR1 expression levels (Figure 6B,C, *P* < 0.05). NCTC 1469 cells with transfection of miR‐92a mimic had up‐regulated expression level of ApoM while cells transfected with miR‐92a inhibitor had down‐regulated expression level of ApoM (Figure 6D,E, *P* < 0.05). Collectively, miR‐92a can promote the expression level of ApoM. NCTC 1469 cells co‐transfected with miR‐92a mimic and LV‐TUG1 had decreased expression level of ApoM when compared with cells transfected with miR‐92a mimic alone (Figure 6D,E, *P* < 0.05), suggesting TUG1 could confront miR‐92a to regulate ApoM expression. Taken together, TUG1 inhibits miR‐92a and enhances FXR1 to down‐regulate ApoM expression level in NCTC 1469 cells.

### 3.7 TUG1 accelerates disease progression of ApoE−/− AS mice

**FIGURE 7:**
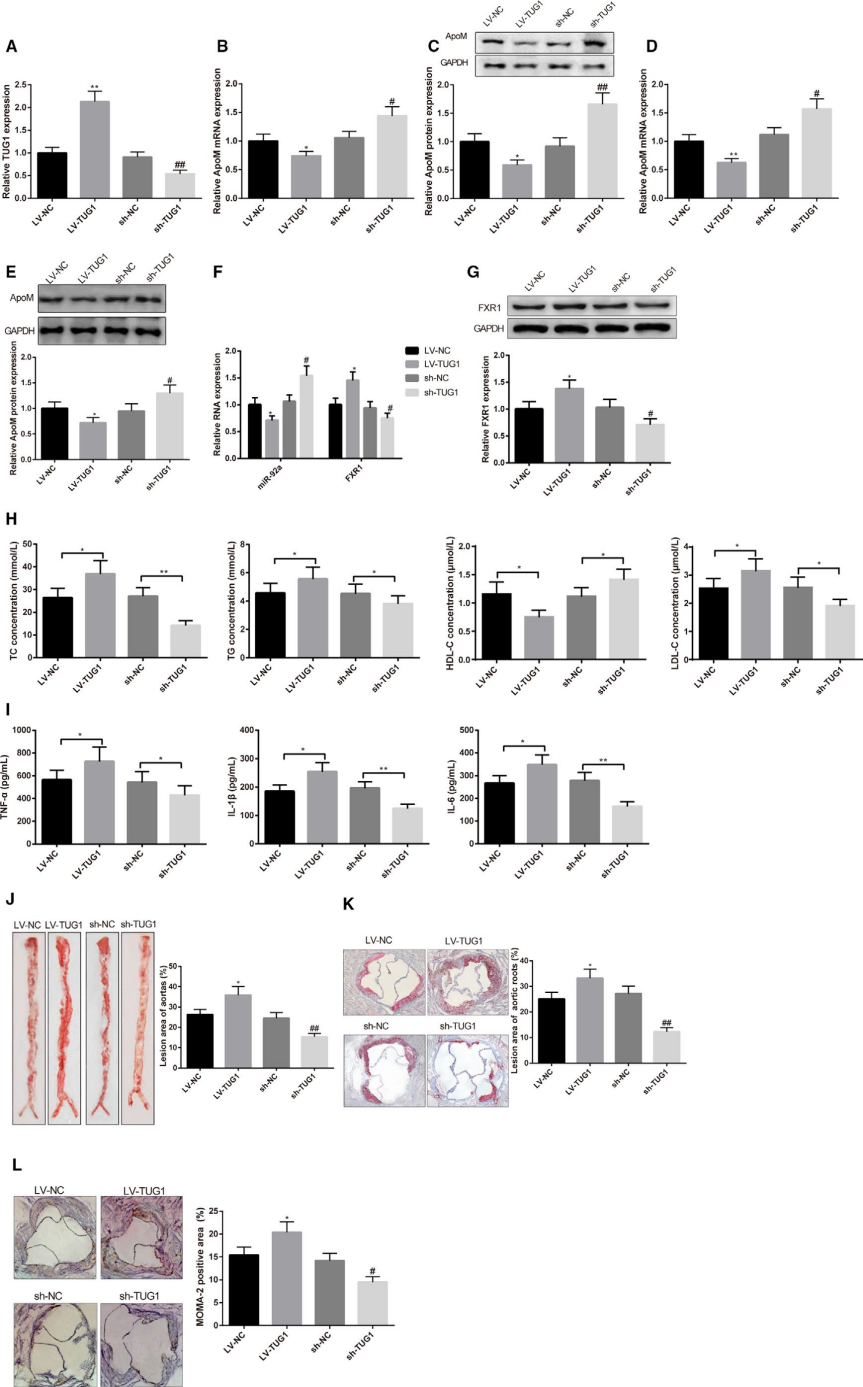
TUG1 deteriorates atherosclerosis (AS) progression in ApoE−/− mice through inhibiting the expression level of ApoM. After mice were injected with LV‐TUG1, sh‐TUG1 or negative control, qRT‐PCR and Western blot were applied to measure the expression level of TUG1 and ApoM in liver tissues or in plasma. TUG1 in liver tissues determined by qRT‐PCR (A), n = 6; mRNA and protein expression of ApoM in liver tissues were measured (B, C), n = 6; mRNA and protein expression of ApoM in plasma were measured (D, E), n = 6; qRT‐PCR (F) and Western blot (G) were used to measure the expression of miR‐92a and FXR1 in liver tissues of mice, n = 6; concentrations of TC, TG, HDL‐C and LDL‐C were detected by kits (H), n = 6; ELISA was utilized to detect the concentrations of TNF‐α, IL‐1β and IL‐6 (I), n = 6; oil red O staining detected the plaque area in aorta (J) and arterial wall (K), n = 6. Immunohistochemistry detected the expression level of MOMA‐2 in aorta root (L), n = 6. TC, cholesterol; TG, triglyceride; LDL‐C, low‐density lipoprotein cholesterin; HDL‐C, high‐density lipoprotein cholesterol; *, compared with LV‐NC group, *P* < 0.05; **, compared with LV‐NC group, *P* < 0.01; # compared with sh‐NC group, *P* < 0.05; ##, compared with sh‐NC group, *P* < 0.01

The effect of TUG1 in AS was then verified in male ApoE−/− AS mice. After mice were injected with LV‐TUG1, the expression level of TUG1 in liver tissues was up‐regulated by 2.13 times (Figure 7A, *P* < 0.01). LV‐TUG1 could decrease the mRNA expression of ApoM by 0.74 times and the protein expression by 0.59 times in liver tissues (Figure 7B,C, *P* < 0.05). The detection of ApoM in plasma showed that mRNA expression of ApoM was decreased by 0.63 times and the protein expression by 0.72 times (Figure 7D,E, *P* < 0.05). The expression of miR‐92a in liver tissues was decreased by 0.71 times after LV‐TUG1 injection (Figure 7F, *P* < 0.05). After mice were injected with LV‐TUG1, the mRNA expression of FXR1 was increased by 1.45 times and the protein expression of FXR1 increased by 1.38 times (Figure 7F,G, *P* < 0.05). LV‐TUG1 injection in ApoE−/− mice could increase TC, TG, LDL‐C and inflammatory cytokines (TNF‐α, IL‐1β and IL‐6), and decrease HDL‐C (Figure 7H,I, *P* < 0.05). Then, oil red O staining on slices demonstrated that plaque area of arterial wall (33.24 ± 3.66% vs 25.14 ± 2.65%) and aorta (35.22 ± 5.28% vs 26.81 ± 2.54%) were enlarged in ApoE−/− mice with LV‐TUG1 injection compared with LV‐NC group (Figure 7J,K, *P* < 0.05). Then, macrophage in aorta was measured using immunohistochemistry. The results revealed that mice with injection of LV‐TUG1 had larger MOMA‐2‐positive area than that in LV‐NC group (20.43 ± 2.37% vs 15.46 ± 1.83%, Figure 7L, *P* < 0.05). Collectively, overexpression of TUG1 inhibits the expression level of ApoM to deteriorate AS progression in ApoE−/− mice, while inhibition of TUG1 can attenuate AS.

## 4 DISCUSSION

Data in this study demonstrate the promotive role of TUG1 in AS progression by inhibiting ApoM expression levels as well as blocking the RCT in macrophages mainly through miR‐92a/FXR1 axis. As a result, knock‐down of TUG1 markedly protects against the progression of AS and promotes CE rate. Importantly, inhibition of TUG1 also increases the expression levels of ABCA1 and ABCG1 in C57BL/6J mice, both of which are critical genes involved in pathogenesis of AS. TUG1 can compete with FXR1 to bind with miR‐92a. Thus, our current works suggest that TUG1 can serve as a promising target for the prevention and treatment of AS.

High‐density lipoprotein is a critical effector in RCT which facilitates the transport of cholesterol from cells in the vessel wall to the liver to maintain balance of cholesterol in arterial wall.^26^ Meanwhile, HDL is one of the endogenous factors that restores the optimal endothelial function in vascular disease and plasma apolipoprotein M‐containing HDL (ApoM + HDL) can activate the G protein‐coupled sphingosine 1‐phosphate (S1P) receptors to promote vascular barrier function.^27^ The CE from macrophage foam cells requires the participation of both ABCA1 and preβ migrating HDL.^28^ Evidence from previous study supported that ApoM are closely associated with HDL.^29^ Furthermore, ApoM also has certain role to play in the formation of the pre‐HDL particles to exert its anti‐atherogenic functions.^30^ It is well known that ApoM is a specific S1P chaperone, whose overexpression can contribute to the stimulation and formation of apoM/S1P complex in HDL by increasing the synthesis and secretion of its cargo, S1P.^31^ In vitro experiments in current study found down‐regulated expression level of ApoM in liver tissues and plasma of HFD mice. In addition to that, we found that the abnormal lipids metabolism also associated with the abnormal expression of TUG1, which was highly expressed in liver tissues of mouse. Those results are consistent with the promotive role of TUG1 in AS reported in previous studies.^13^, ^32^, ^33^ To further explore the possible role and effect of TUG1 on ApoM and AS, we then applied TUG1 gain and loss of function in C57BL/6J mice. As respect, overexpression of TUG1 could reduce the expression of ApoM and inhibit the CE rate in macrophages by mediating the expressions of ABCA1 and ABCG1. In addition to that, anti‐inflammatory cytokines have been highlighted as novel therapeutic targets for AS.^34^ To better elucidate the role of TUG1 in AS, we also measured the plasma expressions of TNF‐α, IL‐1β and IL‐6 in mice injected with LV‐TUG1. The results obtained from ELISA demonstrated that TUG1 could enhance inflammatory response in plasma. According to the recommendation of a previous study,^35^ we also measured with the alternation on TC, TG, LDL‐C and HDL‐C in ApoE−/− after LV‐TUG1 injection, with the conclusion consistently supporting that TUG1 in liver tissue is associated with AS progression. As far as we can see that both TUG1 and ApoM have certain roles to play in AS, but whether TUG1 can regulate ApoM or the other way around to mediate AS is far from elucidated.

Dual luciferase reporter assay and RIP assay revealed that TUG1 can negatively target miR‐92a to mediate FXR1 expressions. To verify this result, we measured the expression levels of miR‐92a and FXR1 in mice injected with TUG1 overexpression and knock‐down plasmid. Interestingly, the data obtained that overexpression of TUG1 can inhibit miR‐92a while promote FXR1 in liver tissue of mice, while reverse results were observed in mice transfected with sh‐TUG1. TUG1 can compete with FXR1 for interaction with miR‐92a. In addition to that, knowledge obtained from other study supported the negative regulatory of FXR1 on ApoM.^24^ Moreover, we verify the regulation of miR‐92a/FXR1 on ApoM in NCTC 1469 cells. Our results showed that miR‐92a can positively regulate the expression level of ApoM. The regulation of TUG1 on ApoM can be counteracted by miR‐92a, and the effect of miR‐92a on ApoM can also be demolished by FXR1, suggesting that TUG1 can inhibit miR‐92a to promote FXR1, so as to down‐regulate the expression level of ApoM. Therefore, TUG1 regulates ApoM expressions in AS mainly through miR‐92a/FXR1 axis. Similarly, up‐regulation of TUG1 was found in endothelial cells induced by high‐dose glucose to enhance cell migration and viability through Wnt pathway and enhance the progression and deterioration of diabetic AS.^32^ Animal models are perfect tools for AS research. We used male ApoE−/− mice to unearth the effect of TUG1 in AS. Consistent with results in our previous observation, LV‐TUG1 can contribute to the down‐regulation of ApoM, enlarged plaque area and increased macrophages in mice. Here, overexpression of TUG1 could suppress the expression of ApoM to promote the progression of AS in mouse.

In conclusion, we have revealed that TUG1 is a potential target for AS. The mechanism of TUG1 deteriorating AS is through miR‐92a/FXR1 axis to inhibit the expression of ApoM and inhibit its anti‐atherogenic effect.

## CONFLICT OF INTEREST

The authors declare they have no conflict of interest.

## AUTHOR CONTRIBUTION

**Liu Yang:** Resources (lead); Supervision (lead); Validation (lead); Writing‐original draft (lead); Writing‐review & editing (lead). **Tie Li:** Resources (lead); Software (lead); Supervision (lead); Validation (lead); Writing‐review & editing (lead).

## Data Availability

The data sets used or analysed during the current study are available from the corresponding author on reasonable request.
